# Medetomidine-vatinoxan-methadone and acepromazine-methadone: comparison of sedative and cardiovascular properties as a preanaesthetic medication in healthy dogs

**DOI:** 10.1186/s13028-025-00844-3

**Published:** 2025-12-02

**Authors:** Vuokko Pekkola, Ira Kallio-Kujala, Marja Raekallio, Jaan Lepajõe, Kati Salla

**Affiliations:** 1 Veterinary Teaching Hospital, Faculty of Veterinary Medicine, University of Helsinki, PO BOX 57, Helsinki, FI-00014 Finland; 2 Department of Equine and Small Animal Medicine, Faculty of Veterinary Medicine, University of Helsinki, PO BOX 57, Helsinki, FI-00014 Finland

**Keywords:** Alpha-2-adrenoceptor agonists, Hypotension, MK-467, Premedication, Sedation

## Abstract

**Background:**

Medetomidine-vatinoxan is a relatively new medicinal product indicated for sedation of healthy dogs. Vatinoxan alleviates medetomidine-induced bradycardia and peripheral vasoconstriction in dogs, but when used as a preanaesthetic medication, it has been shown to cause more hypotension during general anaesthesia compared to medetomidine alone. Our aim was to compare medetomidine-vatinoxan to acepromazine when used as a preanaesthetic medication in a randomised, blinded, clinical study. Healthy client-owned dogs (*n* = 25) scheduled for elective ovariectomy were randomly assigned to receive 0.2 mg/kg intramuscular methadone combined with either 0.01 mg/kg medetomidine and 0.2 mg/kg vatinoxan (group MV, *n* = 13) or 0.02 mg/kg acepromazine (group A, *n* = 12). A sedation scale (SS, range 0–12) and visual analogue scale (VAS, range 0–100 mm) were applied to assess sedation every 5 min until one of the following endpoints was reached: the SS was ≥ 6 or30 min from treatment had passed. After this, general anaesthesia was induced with propofol and maintained with sevoflurane vaporised in oxygen. The need for cardiovascular interventions according to current guidelines was recorded. Statistical comparisons were performed with Student’s t test, the Mann‒Whitney U test and Fisher’s exact test. P-values < 0.05 were considered statistically significant.

**Results:**

The median (range) time to achieve an SS ≥ 6 was 5 (5–10) minutes in the MV group and 20 (10–25) minutes in the A group (P-value < 0.001). The number of dogs needing interventions for hypotension, bradycardia and/or bradyarrhytmias (7 in group MV, 8 in group A) did not significantly differ between the groups.

**Conclusions:**

When used as a preanaesthetic medication in combination with methadone, medetomidine-vatinoxan causes faster onset of sedation, without statistically significant differences in cardiovascular interventions, compared to acepromazine.

**Supplementary Information:**

The online version contains supplementary material available at 10.1186/s13028-025-00844-3.

## Background

 The use of sedative drugs as premedication prior to general anaesthesia is common practice in clinical veterinary medicine. It offers several benefits, including decreased stress in the animal, a reduced need for anaesthetic drugs for the induction and maintenance of anaesthesia and possibly the provision of pre-emptive analgesia [1]. However, commonly used sedative drugs also have undesirable side effects, of which cardiovascular depression is often considered to be the most significant. This is particularly true with alpha-2-adrenoceptor agonist drugs, such as medetomidine and its active enantiomer, dexmedetomidine [2, 3].

Alpha-2-adrenoceptor agonist drugs activate alpha-2-adrenoceptors in the central nervous system, causing sedation, analgesia and sympatholysis. They also bind to alpha-2-adrenoceptors outside the central nervous system, causing, for example, peripheral vasoconstriction, reflex bradycardia and a decreased cardiac index. To overcome these peripherally mediated side effects, (dex)medetomidine can be combined with vatinoxan, which is an alpha-2-adrenoceptor antagonist [4–8]. Unlike the commonly used alpha-2-adrenoceptor antagonist atipamezole, vatinoxan does not cross the blood–brain barrier [9, 10]. When vatinoxan is co-administered intramuscularly with (dex)medetomidine, the sedative properties of (dex)medetomidine remain nearly unchanged, but peripheral vasoconstriction and decreases in heart rate and cardiac index are alleviated [11, 12].

Acepromazine is another commonly used preanaesthetic drug in veterinary medicine. Its sedative properties are mediated through dopaminergic, adrenergic and serotonergic pathways in the central nervous system. Acepromazine causes long-lasting, typically mild or moderate sedation in dogs and a decreased cardiac index, leading to decreased arterial blood pressure [13, 14]. Unlike alpha-2-adrenoceptor agonists, acepromazine has no analgesic properties; thus, it is commonly combined with opioids [15].

In previous studies, there have been conflicting results regarding the depth of sedation when (dex)medetomidine and acepromazine were compared. Several studies have reported no statistically significant differences in sedation scores when dogs are sedated with either acepromazine or (dex)medetomidine with or without an opioid in combination [16–18] whereas in other studies, (dex)medetomidine caused a significantly deeper plane of sedation than did acepromazine [19, 20]. (Dex)medetomidine as a preanaesthetic medication has been linked with a lower incidence of perianaesthetic hypotension than acepromazine [21]. If (dex)medetomidine is combined with vatinoxan, the incidence of hypotension during general anaesthesia increases significantly [22–24]. In a previous experimental study, beagle dogs had lower mean arterial blood pressures (MAP) during general anaesthesia after premedication with acepromazine and butorphanol compared to medetomidine and vatinoxan [25].

The primary aim of our study was to compare the sedative properties of intramuscular (IM) medetomidine-vatinoxan-methadone and acepromazine-methadone in healthy female dogs. Our hypothesis was that dogs treated with medetomidine-vatinoxan-methadone would achieve the desired degree of sedation faster than dogs treated with acepromazine-methadone. Our secondary aim was to compare the incidences of bradycardia and hypotension during general anaesthesia in dogs premedicated with medetomidine-vatinoxan-methadone or acepromazine-methadone.

## Methods

### Animals

The Finnish Medicines Agency (Fimea) was notified before recruitment, and ethical approval was obtained from the University of Helsinki Research Ethics Committee (12/2022). A power analysis (using an online statistical calculator, http://statulator.com) revealed that 12 dogs per group were needed to detect a 10-minute difference in sedation time (standard deviation [SD] 8.5 min). Healthy female dogs aged 6 months to 8 years, weighing 5–50 kg, with an American Society of Anesthesiologists (ASA) physical status classification of I or II, and scheduled for elective ovariectomy could be included for the study. The exclusion criteria included confirmed or suspected pregnancy, history of adverse drug reactions to any of the drugs in the study protocol, and aggressive behaviour. All dogs underwent a preoperative clinical evaluation, including a complete physical examination but no further diagnostic tests, and written informed owner consent was obtained.

### Study design

Dogs were randomly assigned to two treatment groups: group MV (medetomidine 0.01 mg/kg, vatinoxan 0.2 mg/kg [Zenalpha, Vetcare, Helsinki, Finland], and methadone 0.2 mg/kg [Insistor, VetViva Richter, Wels, Austria] IM) and group A (acepromazine 0.02 mg/kg [Plegicil, Pharmaxim, Landskrona, Sweden], and methadone 0.2 mg/kg IM). The choices of drug doses were based on the clinical experience of the authors. Dogs were stratified into three weight classes (5–14.9 kg, 15–29.9 kg and 30–50 kg), and a block design was used to ensure a relatively even distribution of all weight classes in both treatment groups. Randomisation lists were generated with an online tool (http://randomization.com*).*

Since the acepromazine formulation had a concentration of 10 mg/mL, it was diluted with physiological saline at a ratio of 1:9 for precise dosing. Methadone was combined in the same syringe with either diluted acepromazine or medetomidine and vatinoxan, and the syringe was covered with opaque tape to mask the colour of its contents. The total injection volume was 0.04 mL/kg in both treatment groups. Drug preparation was performed by one of the anaesthetists (KS, IKK or JL) who were not assessing sedation. Covering of the syringe was performed as a precaution to ensure blinding since the blinded person was in the same room when drugs were injected into the dog.

### Sedation and anaesthesia

Drugs were injected into the quadriceps femoris muscle. Sedation was assessed every five minutes with a short form of the validated multidimensional sedation scale [26], (Table 1) and visual analogue scale (VAS). We chose the cut-off value for the sedation score (SS) based on a small number of pilots. A score of 6/12 represented the depth of sedation, which was subjectively sufficient to allow intravenous (IV) cannula placement and preoxygenation with a facemask without excessive stress to the dog. Once the SS reached the chosen cut-off value or once 30 min from premedication was passed, the monitoring period for sedation ended. Heart rate (HR) and respiratory rate (RR) were then measured via auscultation of the chest with a stethoscope, and MAP was measured noninvasively with an oscillometric device (petMAP graphic II, Ramsey Medical, Inc., Tampa, Florida, USA) with an appropriately sized cuff placed immediately proximal to the carpus. Most dogs were in sternal recumbency during these measurements.

**Table 1:** The sedation scale

Score	0	1	2	3	4
Spontaneous posture	Standing	Tired but standing	Lying but able to rise	Lying but difficulty rising	Unable to rise
Eye position	Central	Rotated forwards/ downwards but not obscured by third eyelid	Rotated forwards/ downwards and obscured by third eyelid		
Response to noise (clicker sound)	Normal startle reaction (head turn towards noise/ cringe)	Reduced startle reaction (reduced head turn/ minimal cringe)	Minimal startle reaction	Absent reaction	
General appearance/attitude	Excitable	Awake and normal	Tranquil	Stuporous	

Short form of sedation scale published originally by Grint et al. [16] and validated by Wagner et al. [26]. The dog’s posture, eye position, response to noise and general appearance are evaluated and given a score according to a table. The scores are then added to obtain the total sedation score (range 0–12)

After the initial measurements, an IV cannula was placed in the cephalic vein of each dog. Preoxygenation and electrocardiogram monitoring (lead II) were initiated with a multiparameter monitor (Lifescope, Nihon Kohden, Tokyo, Japan). Intravenous fluid therapy (Ringer Lactate AnimalCare, Informed Fluids SRL, Bucharest, Romania) was started at a rate of 5 mL/kg/h with an infusion pump (Infusomat Space, B. Braun Medical, Melsungen, Germany). It was recorded whether the dog tolerated IV cannula placement without the need for manual restraint.

Propofol (Propovet Multidose, Zoetis Animal Health, Parsippany-Troy Hills, New Jersey, USA) was administered as an IV bolus at a dose of 1–2 mg/kg, depending on the level of sedation. A higher dose was used for dogs that were still holding their heads above shoulder level. Anaesthetic depth was evaluated 60 s after the initial bolus by assessing muscle relaxation (particularly jaw tone), eyeball position, and the medial palpebral reflex. Additional IV boluses of 0.5–1 mg/kg were administered every 30–60 s, with repeated evaluation of anaesthetic depth until the dog appeared relaxed, the eyeballs were rotated ventromedially, and the palpebral reflex was absent. Once an adequate depth of anaesthesia was achieved, the trachea was intubated with an appropriately sized cuffed endotracheal tube. The duration of induction was defined as the time from the start of propofol administration to the completion of intubation. The quality of induction and intubation was scored with a simple descriptive scale (Table 2).

**Table 2:** Simple descriptive scale used to score quality of anaesthesia induction and intubation in dogs

Score	Description
0	Smooth induction with easy intubation
1	Slight resistance but smooth, mild cough/swallowing after intubation
2	Mild/moderate resistance, coughing/swallowing, involuntary limb movement
3	Excitement, poor quality of induction, vocalizing, needs manual restraint

Anaesthesia was maintained with sevoflurane (Sevoflo, Zoetis Animal Health, Parsippany-Troy Hills, New Jersey, USA) in 100% oxygen with the initial oxygen flow set to 2–4 L/min and the vaporiser dial 2.5–3%, delivered initially with a small animal anaesthesia machine (Matrx VMS, Midmark, Versailles, Ohio, USA) with a circle anaesthetic breathing system. Dogs were allowed to breathe spontaneously, and end-tidal carbon dioxide tension (ETCO_2_) and arterial blood oxygen saturation (SpO_2_) were monitored with the previously mentioned multiparameter monitor. Maropitant 1 mg/kg (Prevomax, Eurovet Animal Health, Bladel, Netherlands) was administered IV to all dogs during surgical preparation. Cardiovascular data was not recorded to the study sheet for at least five minutes after administration of maropitant. When surgical preparation was finished, 2 mg/kg ropivacaine (Ropivacain Fresenius Kabi 10 mg/mL, Fresenius Kabi, Uppsala, Sweden) was infiltrated subcutaneously into the area of the planned incision, after which the dog was transported to the operating room. The endotracheal tube was connected to the circle breathing system of the anaesthetic workstation (Carestation 650, GE Healthcare, Chicago, Illinois, USA), and in addition to the previously mentioned parameters, the end-tidal fraction of sevoflurane (ET_sevo_), the fraction of inspired oxygen (FIO_2_) and the oesophageal body temperature (T) were monitored continuously. Blood pressure monitoring was continued with the previously mentioned oscillometric device throughout the whole anaesthetic period. All monitored parameters were recorded at five-minute intervals. The dogs were actively warmed with a forced warm air heating device (Bair Hugger, 3M, Saint Paul, Minnesota, USA) to maintain the core body temperature between 37.0 and 38.0 °C. The aim was to achieve an ET_sevo_ of 2.0% prior to making a skin incision. Standard midline open ovariectomy or ovariohysterectomy was performed by 5th year veterinary students, who were supervised by experienced veterinary surgeons. Lidocaine 2% (Lidor, VetViva Richter, Wels, Austria, dose 2 mg/kg) was splashed on top of each ovarian suspensory ligament, after which 60 s was allowed until the ovary was manipulated. When abdominal cavity closure started, 0.2 mg/kg meloxicam (Meloxidyl, Ceva Sante Animale, Libourne, France) was given IV.

**Fig. 1:**
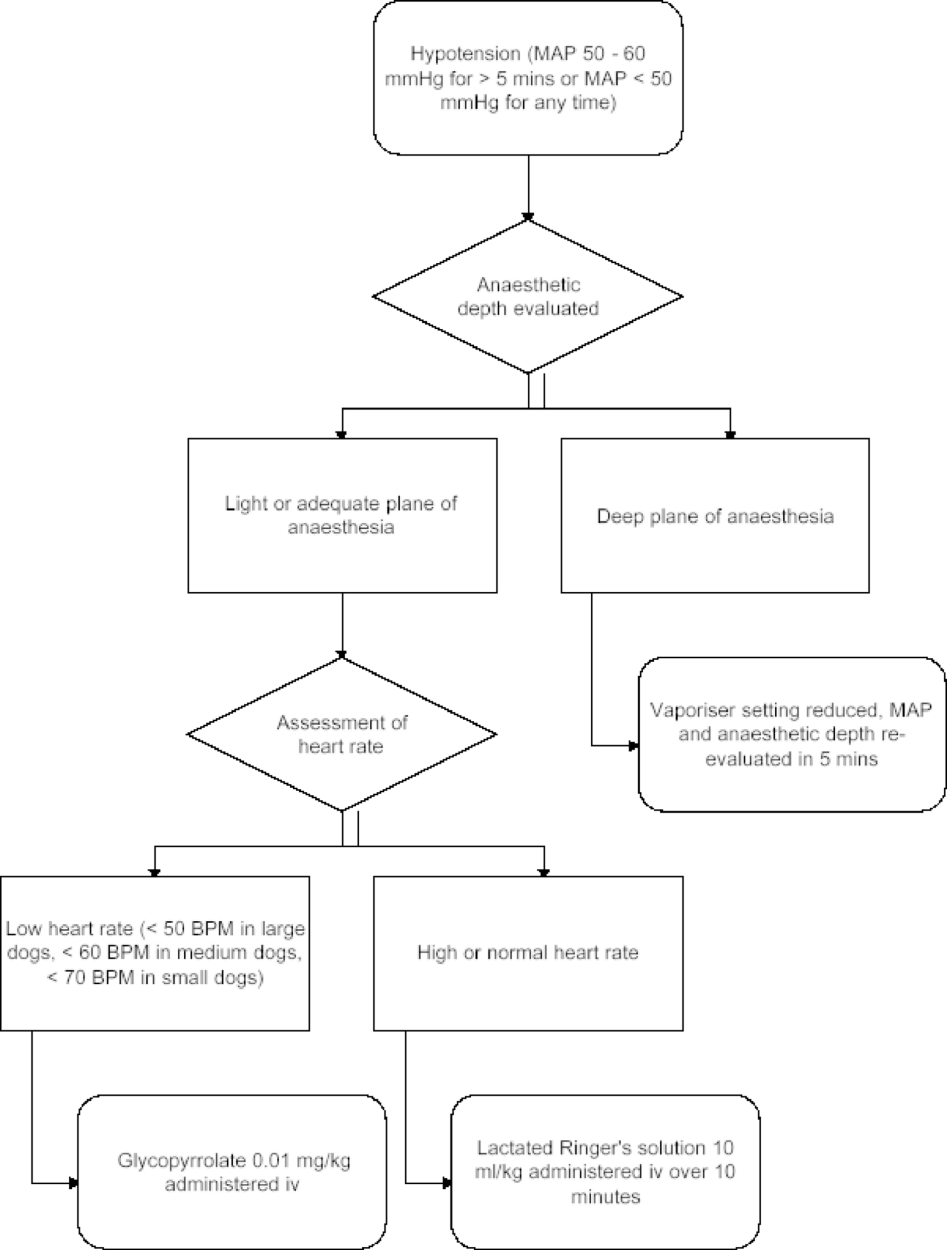
Management of hypotension. Flowchart explaining initial management to arterial hypotension during general anaesthesia in dogs anaesthetised with sevoflurane for elective ovariectomy. MAP = mean arterial pressure, BPM = beats per minute

In the case of hypotension (MAP 50–60 mmHg at two consecutive measurements at five-minute intervals or MAP < 50 mmHg at a single measurement), the depth of anaesthesia was evaluated, and the sevoflurane vaporiser setting was reduced, if possible. MAP and depth of anaesthesia were then re-evaluated in five minutes and if hypotension was not resolved, it was treated. If the dog was bradycardic [HR < 50 beats per minute (BPM) in large dogs; HR < 60 BPM in medium sized dogs; HR < 70 BPM in small dogs] and hypotensive, 0.01 mg/kg glycopyrrolate (Robinul, Meda Pharma, Bad Homburg, Germany) was administered IV. If there was hypotension without bradycardia, a crystalloid fluid bolus (lactated Ringer’s solution) of 10 mL/kg was administered IV over 10 min. Initial approach to hypotension is explained in Fig. 1.

If glycopyrrolate resolved bradycardia but hypotension persisted, fluid bolus was administered as described earlier. If the MAP was still < 60 mmHg after glycopyrrolate and/or fluid bolus, noradrenaline (Noradrenalin Abcur, Abcur, Helsingborg, Sweden) variable rate IV infusion (VRI) was started at a rate of 0.1 µg/kg/min. The dose rate was increased at 0.1 µg/kg/min every 5 min until the MAP was > 60 mmHg. The aim was to maintain the MAP between 60 and 100 mmHg. If the MAP increased above 100 mmHg, the noradrenaline dose rate was decreased at 0.1 µg/kg/min every 5 min.

Mild bradycardia (HR > 40, yet below previously mentioned cut-offs according to size of the dog) with normotension did not necessitate intervention unless there was a sudden drop in HR and/or hemodynamically significant or progressing bradyarrhythmias (such as 2nd degree AV blocks and/or ventricular escape beats) were detected, in which case atropine 0.02 mg/kg (Atropin, Takeda, Tokyo, Japan) was administered intravenously and repeated every 2 min, if necessary, until a response was observed (HR reaching or exceeding 50 BPM and arrhythmias were resolved).

Anaesthetic depth was evaluated clinically on the basis of jaw tone, the medial palpebral reflex and eyeball position. If the depth of anaesthesia was clinically evaluated to be too superficial or too deep, the vaporiser setting was adjusted accordingly. Fentanyl (Fentanyl Hameln, Hameln, Hameln, Germany) at a dose of 2 µg/kg was given as a bolus IV if HR and/or MAP increased > 20% from the values recorded prior to skin incision. This dose was repeated every 2 min, if necessary, until a response was observed (HR and/or MAP decreased and did not increase again when the surgical procedure was continued). Other possible anaesthetic and surgical complications were addressed, as the veterinarian in charge seemed appropriate. All interventions were recorded on the study sheet, and the anaesthetic record.

Once the surgery was finished, the monitoring devices were detached, the sevoflurane vaporiser was turned off, and the fresh gas flow rate was increased to 2–4 L/min. Simultaneously, 0.5 mg/kg propofol was administered IV to prevent excitation in the early recovery phase. Once ET_sevo_ was 0.5% or less, the endotracheal tube was disconnected from the breathing system, and the dog was transported to the recovery room. Dogs were extubated once they had regained their swallowing reflex. Monitoring of HR and RR was continued until the dogs could remain in sternal recumbency. The durations of anaesthesia and recovery were defined as the time between the start of induction and extubation and as the time between the end of sevoflurane delivery and when the dog maintained sternal recumbency, respectively. The quality of recovery was evaluated with a simple descriptive scale (range 0–3, Table 3). Pain was assessed one hour postextubation via the short form of the Glasgow Composite Measure Pain Scale [27], with additional analgesia (buprenorphine 0.02 mg/kg IV, Bupaq Multidose, Richter Pharma, Wels, Austria) given if the pain score was >5/24. The dogs were monitored for at least three hours postextubation before being sent home with prescribed oral meloxicam (Metacam, Boehringer Ingelheim Vetmedica, Ingelheim, Germany) 0.1 mg/kg once a day for three to five days starting on the day following surgery, and paracetamol (Paramax Junior, Vitabalans, Hämeenlinna, Finland) 10–15 mg/kg every 8–12 h, if appeared painful despite the administration of meloxicam. Owners were contacted the following day via telephone, and preset questions were presented (Additional File 1).

**Table 3:** Simple descriptive scale used to score quality of recovery from anaesthesia in dogs

Score	Description
0	Excellent, smooth and fast transition to conscious state
1	Good, mild whining or prolonged sedation, mild and self-limiting limb paddling
2	Fair, not smooth, moderate excitement or prolonged sedation
3	Poor, marked excitement requiring additional sedation or severely prolonged sedation

An experienced veterinary anaesthetist (VP), who was blinded to the treatment groups, evaluated sedation and managed anaesthesia and recovery for all dogs.

### Statistical analysis

Cardiovascular data collected from certain predetermined time points were used for statistical analysis: (1) once the sedation score had reached or exceeded the cut-off value or once 30 min after the injection of premedication had passed, (2) just before the induction of general anaesthesia, (3) just before surgical incision, (4) when the first ovarian vessels were clamped, (5) when the second ovarian vessels were clamped (moments of maximal surgical stimulation) and (6) when the closure of the abdominal cavity had started. In addition, the nadir MAP was recorded for each dog.

All continuous numerical data were tested for normality with the Shapiro‒Wilk test. With normally distributed data (duration of anaesthesia, HR, MAP, ETCO_2_, induction time, time between premedication and start of induction, amount of propofol, duration of recovery), comparisons of means were performed with Student’s t test (two-sided). With non-normally distributed numerical data (age, weight, duration of surgery, time to reach sternal recumbency, time to reach cut-off sedation score, ET_sevo_) and ordinal data (body condition score, sedation score, sedation VAS, pain score), the Mann‒Whitney U test was used for comparisons between groups. Fisher’s exact test was used for comparisons between groups in terms of yes/no type of parameters (tolerance of IV cannula placement, incidence of intraoperative interventions). P-values < 0.05 were considered statistically significant. All the statistical analyses were performed with commercial statistics software (IBM SPSS Statistics Version 29.0.2.0(20), IBM, Armonk, New York, USA). Data are presented as the mean ± SD or median (minimum–maximum). In addition, 95% confidence intervals are presented for the mean differences in cardiovascular parameters.

## Results

A total of 25 dogs were recruited for the study, 13 of which were randomised into group MV and 12 into group A. The study population consisted of several dog breeds with no more than two dogs of each breed. Three of the dogs (one in group MV and two in group A) were chondrodystrophic. In these dogs, it was impossible to obtain a proper fit of the cuff for blood pressure measurements due to shape of the front limb, and the obtained readings were clinically judged to be unrealistically high. For this reason, blood pressure data from these three dogs were excluded from the statistical analysis. No significant differences in demographic data or durations of anaesthesia or surgery were detected between the groups, and the data are presented in Table 4.

**Table 4:** Demographic and procedure duration data for dogs anaesthetised for elective ovariectomy

Parameter	Group MV (*n* = 13)	Group A (*n* = 12)	*P*-value
Age (months)	30 (11–95)	23.5 (14–68)	0.695
Weight (kg)	14.5 (5.8–34.1)	14.7 (6.2–35.2)	1.000
Body condition score	5/9 (4–7)	5/9 (4–6)	0.205
Duration of anaesthesia (min)	168 ± 13	171 ± 14	0.595
Duration of surgery (min)	121 (96–125)	119 (101–128)	0.695

Dogs were premedicated with methadone 0.2 mg/kg IM combined with either medetomidine 0.01 mg/kg and vatinoxan 0.2 mg/kg (group MV) or acepromazine 0.02 mg/kg (group A). Anaesthesia was induced with IV propofol and maintained with sevoflurane. Data are presented as mean ± SD for duration of anaesthesia and median (range) for other parameters

**Fig. 2:**
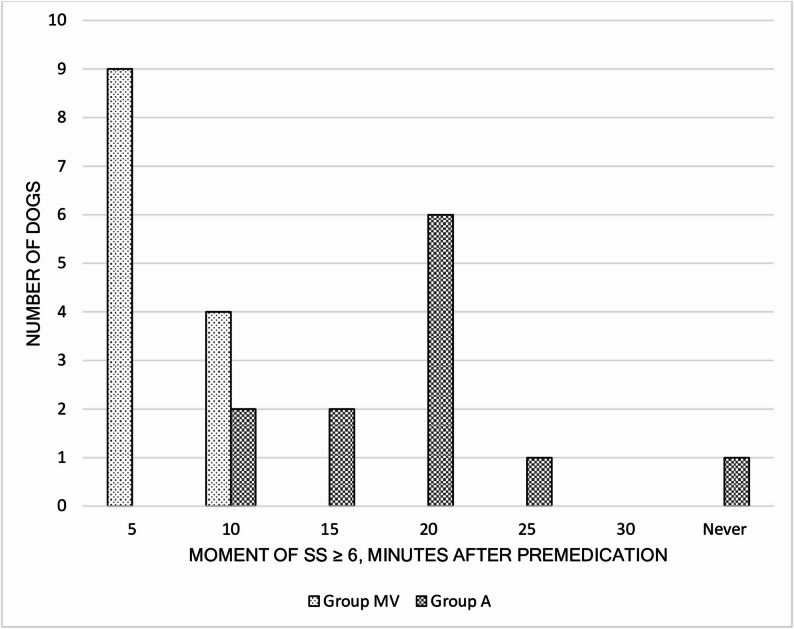
Timepoints (after premedication) when desired sedation was achieved. Distribution of timepoints when dogs premedicated with either medetomidine 0.01 mg/kg, vatinoxan 0.2 mg/kg and methadone 0.2 mg/kg (Group MV) IM or acepromazine 0.02 mg/kg and methadone 0.2 mg/kg (Group A) IM reached or exceeded chosen cut-off value (6/12) in sedation scores (SS), indicating sufficient sedation for IV cannula placement and preoxygenation with a facemask. The difference between groups in amount of time needed to achieve desired sedation is statistically significant

Sedation scores were significantly greater in group MV than in group A at 5 min after premedication. The time to reach or exceed the predetermined cut-off value for the SS was significantly shorter in group MV than in group A (Fig. 2), as was the time between the injection of premedication and the start of anaesthesia induction. Compared with dogs in group A, those in group MV were more likely to tolerate IV cannula placement without manual restraint (Table 5). Most dogs in group MV reached or exceeded the cut-off value for the SS already at 5 min; for this reason, it was not possible to compare the SS between groups beyond this timepoint. The last recorded SS for each dog (final SS) was significantly greater in group MV than in group A. One dog in group A did not reach an SS ≥ 6 by 30 min.

**Table 5:** Sedation data

Parameter	Group MV (*n* = 13)	Group A (*n* = 12)	*P*-value
SS baseline	0 (0–1)	0 (0–1)	0.852
SS 5 min	7 (5–11)	2.5 (0–3)	< 0.001
Final SS	8 (6–11)	6 (3–7)	< 0.001
Sedation VAS baseline	0 (0–0)	0 (0–0)	1.000
Sedation VAS 5 min	39 (30–56)	13.5 (4–27)	< 0.001
Final sedation VAS	48 (36–70)	33 (9–60)	< 0.001
Timepoint when SS ≥ 6 (min)	5 (5–10)	20 (10–25)	< 0.001
Time to reach sternal recumbency (s)	206 (58–510)	351.5 (73–994)	0.022
Time between premedication and induction start (min)	33 ± 5.5	41 ± 6.0	0.001
Tolerance for IV cannula placement without restraint (yes/no)	10 / 3	2 / 10	0.005

Sedation scores (SS), sedation visual analogue scales (VAS) in mm, timepoint when SS reached or exceeded agreed cut-off value, time for dog to lay in sternal recumbency, time between premedication and start of anaesthesia induction, and tolerance of intravenous (IV) cannula placement in dogs premedicated with methadone 0.2 mg/kg combined with either medetomidine 0.01 mg/kg and vatinoxan 0.2 mg/kg (group MV) IM or acepromazine 0.02 mg/kg (group A) IM. Final SS and final sedation VAS refer to last recorded SS and sedation VAS of each dog. Data are presented as median (minimum – maximum) or mean ± standard deviation

HR was significantly lower in group MV than in group A during sedation (*P* = 0.001) but not at the other reference points. No significant differences in MAP or the number of dogs needing intervention to maintain cardiovascular stability were detected between the groups (Tables 6 and 7). In 6 dogs (2 in the MV group and 4 in the A group) hypotension was accompanied with bradycardia and treated successfully with glycopyrrolate. In 4 dogs (2 in each group), hypotension was treated successfully with a fluid bolus. One dog in group MV needed both glycopyrrolate and a fluid bolus for the correction of hypotension, and one dog in each group eventually needed noradrenaline VRI to maintain an acceptable MAP.

**Table 6:** Heart rates

Parameter	Group MV (*n* = 13)	Group A (*n* = 12)	Mean difference between groups	95% confidence interval for mean difference		*P*-value
				Lower	Upper	
HR baseline	114 ± 16	114 ± 19	0	-14	15	0.945
HR sedation	59 ± 11	90 ± 24	-30	-46	-14	0.001
HR before induction	66 ± 29	86 ± 24	-20	-42	2	0.073
HR before incision	80 ± 21	81 ± 22	-1	-19	17	0.887
HR 1st ovarian vessels clamped	91 ± 25	95 ± 24	-4	-24	16	0.708
HR 2nd ovarian vessels clamped	86 ± 16	95 ± 13	-9	-21	3	0.141
HR abdominal closure started	83 ± 13	87 ± 11	-4	-14	6	0.433
Bradyarrhytmias detected (yes/no)	3/10	2/10	N/A	N/A	N/A	1.000

Heart rates (HR, beats per minute) measured at pre-determined reference points and incidence of bradyarrhytmias (2nd degree atrioventricular blocks or ventricular escape beats) in dogs anaesthetised with sevoflurane for elective ovariectomy. Dogs were premedicated with methadone 0.2 mg/kg combined with either medetomidine 0.01 mg/kg and vatinoxan 0.2 mg/kg (group MV) IM or acepromazine 0.02 mg/kg (group A) IM. Anaesthesia was induced with IV propofol. Data are presented as mean ± standard deviation for HR

**Table 7:** Mean arterial blood pressures

Parameter	Group MV (*n* = 12)	Group A (*n* = 10)	Mean difference between groups	95% confidence interval for mean difference		*P*-value
				Lower	Upper	
MAP sedation	118 ± 24	100 ± 19	18	-1	37	0.063
MAP before induction	96 ± 23	97 ± 20	-1	-20	18	0.924
MAP before incision	70 ± 11	78 ± 21	-8	-23	8	0.320
MAP 1st ovarian vessels clamped	87 ± 32	86 ± 15	1	-21	23	0.919
MAP 2nd ovarian vessels clamped	84 ± 14	85 ± 12	0	-12	11	0.934
MAP abdominal closure started	86 ± 15	79 ± 9	7	-4	17	0.206
MAP nadir	59 ± 9	59 ± 4	0	-6	6	0.941
Hypotension detected (yes/no)	6/6	7/3	N/A	N/A	N/A	0.415

Mean arterial blood pressures (MAP, mmHg) measured with oscillometric device at pre-determined reference points, and incidence of hypotension (MAP 50–60 mmHg for > 5 min or MAP < 50 mmHg for any time) in dogs anaesthetised with sevoflurane for elective ovariectomy. Dogs were premedicated with methadone 0.2 mg/kg combined with either medetomidine 0.01 mg/kg and vatinoxan 0.2 mg/kg (group MV) IM or acepromazine 0.02 mg/kg (group A) IM. Anaesthesia was induced with IV propofol. Data are presented as mean ± standard deviation for MAP

The durations of induction and recovery were significantly shorter in the dogs in group MV than in those in group A. The amount of propofol needed for induction was also lower, and the quality of induction was better in group MV than in group A (Table 8). Data regarding gastrointestinal complications and other owner-reported deviations from normal behaviour are reported in Additional File 2, and respiratory parameters, intraoperative fentanyl administration and postoperative pain scores are presented in Additional File 3. None of the dogs needed buprenorphine postoperatively.

**Table 8:** Anaesthesia induction and recovery data in dogs anaesthetised with Sevoflurane for elective ovariectomy

Parameter	Group MV (*n* = 13)	Group A (*n* = 12)	*P*-value
Duration of induction (min)	4.8 ± 0.8	6.3 ± 2.0	0.025
Quality of induction (0–3)	0 (0–1)	1 (0–3)	0.039
Amount of propofol (mg/kg)	2.4 ± 0.5	4.8 ± 1.4	< 0.001
Duration of recovery (min)	8.9 ± 3.3	14.2 ± 6.0	0.015
Quality of recovery (0–3)	1 (0–2)	1 (0–2)	0.539

Dogs were premedicated with methadone 0.2 mg/kg combined with either medetomidine 0.01 mg/kg and vatinoxan 0.2 mg/kg (group MV) IM or acepromazine 0.02 mg/kg (group A) IM. Anaesthesia was induced with IV propofol. Continuous normally distributed data are presented as mean ± standard deviation and categorical data as median (minimum – maximum). In quality of induction and recovery, 0 represents the best score and 3 the worst

## Discussion

Our results supported our hypothesis that dogs sedated faster in group MV than in group A. Our hypothesis and sample size calculation were not built to compare the depth of sedation between groups, but our results suggest that within a given time frame, deeper sedation is achieved with medetomidine-vatinoxan-methadone than with acepromazine-methadone. This finding has several clinical and practical implications. First, deep sedation in dogs improves the safety of the personnel working with them, especially if the dog is nervous or even aggressive. Animal bites are among the most common causes of work-related injuries in veterinarians and veterinary support staff, and sedation could prevent significant amounts of bites [28]. Second, deeper sedation results in less stress for the animal, which could potentially improve anaesthetic safety since acute stress increases the likelihood of acute cardiovascular events [29]. Finally, predictable onset and depth for sedation and faster recovery from general anaesthesia improve patient flow and promote easy planning of the surgical schedule.

In our study, the quality of induction was better, and the duration of induction was slightly shorter in group MV than in group A. The amount of propofol used was markedly lower in group MV than in group A, which is consistent with the known anaesthetic-sparing effect of (dex)medetomidine [20, 30, 31]. A lower amount of propofol for the induction of anaesthesia has been linked to a lower incidence and shorter duration of postinduction apnoea [32].

The incidence of hypotension was high in our study, as reported repeatedly in earlier studies in which dogs were anaesthetised with volatile agents [24, 25, 33, 34], highlighting the need for blood pressure monitoring during general anaesthesia, even in completely healthy dogs. Although we did not see a difference in cardiovascular parameters between groups, it must be recognized that the number of dogs in our study was small, and the calculated 95% confidence intervals for the mean differences in HR and MAP suggest that it might be possible to obtain clinically significant differences in these parameters with larger sample sizes. The incidence of hypotension in group MV was in line with other studies where medetomidine and vatinoxan were used in dogs anaesthetised with sevoflurane [22, 24]. Hypotension during general anaesthesia may decrease vital organ perfusion, potentially leading to severe complications, including acute kidney injury [35, 36]. We managed hypotension in a stepwise manner, starting from simple interventions such as reducing vaporiser setting, correcting bradycardia with glycopyrrolate, or administering intravenous crystalloid fluid boluses, and proceeded to noradrenaline VRIs only if they were not effective. Noradrenaline was our vasoactive drug of choice because of earlier results showing that both MAP and cardiac output increased when noradrenaline was infused into dogs treated with medetomidine, vatinoxan and isoflurane [37]. In this study, we managed to treat hypotension during general anaesthesia in all dogs who required treatment. However, one dog in each group required noradrenaline VRI to maintain acceptable blood pressure during general anaesthesia. Hypotension, which is refractory to treatment with intravenous fluids and anticholinergic drugs, can pose a challenge to a general practitioner who does not have access to syringe pumps or vasoactive drugs.

Our study had several limitations. Sedation assessment is subjective, and the limited published data on sedation scoring make comparisons between studies difficult. In addition to using the only validated scoring system [26], we evaluated sedation with a visual analogue scale and timed the appearance of certain signs of sedation. Different methods were used for assessment since they have different strengths and limitations: the multidimensional scale considers different signs of sedation, but it is more rigid than the VAS, which is more flexible but also more subjective. All these parameters similarly indicated significantly faster and deeper sedation in group MV than in group A. The differences between groups in terms of sedation were so obvious that blinding was compromised for general anaesthesia and recovery. The clinical nature of the study meant that we could not perfectly standardise the sedation conditions for all dogs. We gave it our best effort, however, by sedating the dogs in an empty ward with no other patients or people moving in the room. No significant differences in baseline sedation scores were detected between the groups. The cut-off time of 30 min for our sedation period was dictated by the surgical schedule. However, only one dog in group A failed to achieve the cut-off sedation score within 30 min. The doses of drugs we chose were based on our clinical experience and were relatively low compared to some other studies or general recommendations reported in the literature [38]. Higher doses of acepromazine and medetomidine might have caused deeper plane of sedation. Our sample size calculation was limited to identifying differences between treatment groups in time to achieve the desired sedation score. For this reason, in other parameters where no significant differences were observed, a type II (false negative) error may have occurred, with failure to identify important differences between treatments. We have provided 95% confidence intervals for the mean differences in HR and MAP to aid interpretation of these results. We used a noninvasive oscillometric device for blood pressure measurements, which, although less precise than invasive blood pressure measurement, is the most commonly used method for blood pressure monitoring in healthy dogs during anaesthesia. The device we used has been shown to yield MAPs that are in relatively good agreement with measurement results obtained from invasive blood pressure monitoring in anaesthetised dogs [39]. The fit of the cuff was challenging in chondrodystrophic dogs, which is why we excluded blood pressure data from three dogs, making our sample size even smaller. Finally, our inclusion and exclusion criteria might have caused selection bias in our study population. Our study population consisted of only female dogs and thus the results may not be applied directly to male dogs. We chose to exclude extremities of size since we dosed medetomidine based on weight and not on body surface area, because we wanted our study protocol to reflect common practice in clinical small animal anaesthesia.

## Conclusions

Using medetomidine-vatinoxan-methadone as a preanaesthetic medication instead of acepromazine-methadone offers benefits, including shorter onset of sedation and recovery times, a deeper plane of sedation and decreased consumption of anaesthetic induction drugs. Hypotension during general anaesthesia was a common complication in both our study groups, emphasizing the importance of blood pressure monitoring and ability to treat hypotension.

## Supplementary Information

Below is the link to the electronic supplementary material.

Supplementary Material 1

Supplementary Material 2

Supplementary Material 3

## Data Availability

The datasets used and/or analysed during the current study are available from the corresponding author on reasonable request.

## Abbreviations

BPM: beats per minute
ETCO_2_: end-tidal carbon dioxide tension
ET_sevo_: end-tidal fraction of sevoflurane
HR: heart rate
IM: intramuscular
IV: intravenous
MAP: mean arterial blood pressure
RR: respiratory rate
SD: standard deviation
SS: sedation score
VAS: visual analogue scale
VRI: variable rate infusion
