# Supplementary material for: Medetomidine-vatinoxan-methadone and acepromazine-methadone: comparison of sedative and cardiovascular properties as a preanaesthetic medication in healthy dogs
Source: Acta Vet Scand. 2025 Dec 2;68:1. doi: 10.1186/s13028-025-00844-3 (PMC12777501; doi:10.1186/s13028-025-00844-3)
Supplement: Supplementary file 1 — Supplementary Material 1 [file 13028_2025_844_MOESM1_ESM.pdf]

Additional file 1. Questions presented to owners of the dogs one day after elective ovariectomy. Questions were originally presented in Finnish and translated to English for publication.

Question	Yes	No
Has the dog been eating with normal appetite?		
Has the dog been drinking as usual?		
Has the dog vomited?		
Has the dog defecated?		
If yes, has the fecal consistency been normal?		
Has the dog urinated as usual?		
Has the dog been more tired as usual on the night of surgery?		
Has the dog been more tired as usual next morning after surgery?		
Has the owner given paracetamol to the dog?		
