# Supplementary material for: Medetomidine-vatinoxan-methadone and acepromazine-methadone: comparison of sedative and cardiovascular properties as a preanaesthetic medication in healthy dogs
Source: Acta Vet Scand. 2025 Dec 2;68:1. doi: 10.1186/s13028-025-00844-3 (PMC12777501; doi:10.1186/s13028-025-00844-3)
Supplement: Supplementary file 2 — Supplementary Material 2 [file 13028_2025_844_MOESM2_ESM.pdf]

Additional file 2. Intra- or postoperative gastrointestinal complications and abnormal behaviour.

Complication	Group MV (n = 13)	Group A (n = 12)
Abnormal appetite	2	2
Abnormal drinking	1	5
Vomiting	0	0
Loose faeces or diarrhoea	2	5
Treated at home	*1*	*3*
Needed veterinary care	*1*	*2*
Abnormal urination	0	0
Tired in the evening	13	12
Tired next morning	5	6
Need for paracetamol	5	3
Gastro-esophageal reflux (fluid visible in oral cavity during anaesthesia)	1	1
Involuntary defecation during sedation	2	0

Numbers of dogs experiencing abnormal behaviour, needing paracetamol and having gastrointestinal disturbances intra- or postoperatively during or after elective ovariectomy. Dogs were premedicated intramuscularily with methadone 0.2 mg /kg combined with either medetomidine 0.01 mg/kg and vatinoxan 0.2 mg/kg (group MV) or acepromazine 0.02 mg/kg (group A), anaesthesia was induced with intravenous propofol and maintained with sevoflurane in oxygen.
