# Supplementary material for: Medetomidine-vatinoxan-methadone and acepromazine-methadone: comparison of sedative and cardiovascular properties as a preanaesthetic medication in healthy dogs
Source: Acta Vet Scand. 2025 Dec 2;68:1. doi: 10.1186/s13028-025-00844-3 (PMC12777501; doi:10.1186/s13028-025-00844-3)
Supplement: Supplementary file 3 — Supplementary Material 3 [file 13028_2025_844_MOESM3_ESM.pdf]

Additional file 3. Respiratory parameters, need for intraoperative rescue analgesia and postoperative pain scores.

Parameter	Group MV (n = 13)	Group A (n = 12)	P-value (two-sided)
$ETCO_2$ (mmHg) before incision	48 ± 4	43±5	0.005
$ETCO_2$ (mmHg) 1st ovarian vessels clamped	46 ± 5	43 ± 5	0.104
$ETCO_2$ (mmHg) 2nd ovarian vessels clamped	46 ± 4	44 ± 3	0.319
$ETCO_2$ (mmHg) abdominal closure started	46 ± 5	45 ± 4	0.564
Mechanical/spontaneous ventilation	1/12	3/9	0.322
ET_{sevo} (%) before incision	2.0 (1.9 – 2.2)	2.1 (1.9 – 2.3)	0.434
ET_{sevo} (%) 1st ovarian vessels clamped	2.1 (1.9 – 2.2)	2.15 (1.9 – 2.3)	0.226
ET_{sevo} (%) 2nd ovarian vessels clamped	2.1 (1.7 – 2.4)	2.1 (1.9 – 2.4)	1.000
ET_{sevo} (%) abdominal closure started	2.05 (1.6 – 2.4)	2.1 (1.9 – 2.5)	0.645
Fentanyl yes/no	4/9	4/8	1.000
Pain score (GCMPS)	3 (0 – 5)	2.5 (1 – 4)	1.000

Dogs were anaesthetised with sevoflurane for elective ovariectomy and premedicated intramuscularily with methadone 0.2 mg/kg combined with either medetomidine 0.01 mg/kg and vatinoxan 0.2 mg/kg (group MV) or acepromazine 0.02 mg/kg (group A) and anaesthesia was induced with IV propofol. $ETCO_2$=end-tidal carbon dioxide tension, ET_{sevo}=end-tidal fraction of sevoflurane, GCMPS=Glasgow composite measure pain scale (short form). Data are presented as mean±SD or median (minimum - maximum).
